# Rapid classification of epidemiologically relevant age categories of the malaria vector, *Anopheles funestus*

**DOI:** 10.1186/s13071-024-06209-5

**Published:** 2024-03-18

**Authors:** Emmanuel P. Mwanga, Doreen J. Siria, Issa H. Mshani, Sophia H. Mwinyi, Said Abbasi, Mario Gonzalez Jimenez, Klaas Wynne, Francesco Baldini, Simon A. Babayan, Fredros O. Okumu

**Affiliations:** 1 Environmental Health and Ecological Sciences Department, Ifakara Health Institute, P.O. Box 53, Morogoro, Tanzania; 2 School of Biodiversity, One Health and Veterinary Medicine, University of Glasgow, Glasgow, G12 8QQ UK; 3 School of Chemistry, University of Glasgow, Glasgow, G12 8QQ UK; 4 School of Public Health, Faculty of Health Sciences, University of the Witwatersrand, Johannesburg, South Africa; 5 School of Life Science and Bioengineering, The Nelson Mandela African Institution of Science and Technology, P. O. Box 447, Arusha, Tanzania

**Keywords:** Malaria, *Anopheles funestus*, Deep learning, Machine learning, Ifakara Health Institute, Mid-infrared spectroscopy

## Abstract

**Background:**

Accurately determining the age and survival probabilities of adult mosquitoes is crucial for understanding parasite transmission, evaluating the effectiveness of control interventions and assessing disease risk in communities. This study was aimed at demonstrating the rapid identification of epidemiologically relevant age categories of *Anopheles funestus*, a major Afro-tropical malaria vector, through the innovative combination of infrared spectroscopy and machine learning, instead of the cumbersome practice of dissecting mosquito ovaries to estimate age based on parity status.

**Methods:**

*Anopheles funestus* larvae were collected in rural south-eastern Tanzania and reared in an insectary. Emerging adult females were sorted by age (1–16 days old) and preserved using silica gel. Polymerase chain reaction (PCR) confirmation was conducted using DNA extracted from mosquito legs to verify the presence of *An. funestus* and to eliminate undesired mosquitoes. Mid-infrared spectra were obtained by scanning the heads and thoraces of the mosquitoes using an attenuated total reflection–Fourier transform infrared (ATR–FT-IR) spectrometer. The spectra (*N* = 2084) were divided into two epidemiologically relevant age groups: 1–9 days (young, non-infectious) and 10–16 days (old, potentially infectious). The dimensionality of the spectra was reduced using principal component analysis, and then a set of machine learning and multi-layer perceptron (MLP) models were trained using the spectra to predict the mosquito age categories.

**Results:**

The best-performing model, XGBoost, achieved overall accuracy of 87%, with classification accuracy of 89% for young and 84% for old *An. funestus*. When the most important spectral features influencing the model performance were selected to train a new model, the overall accuracy increased slightly to 89%. The MLP model, utilizing the significant spectral features, achieved higher classification accuracy of 95% and 94% for the young and old *An. funestus*, respectively. After dimensionality reduction, the MLP achieved 93% accuracy for both age categories.

**Conclusions:**

This study shows how machine learning can quickly classify epidemiologically relevant age groups of *An. funestus* based on their mid-infrared spectra. Having been previously applied to *An. gambiae*, *An. arabiensis* and *An. coluzzii*, this demonstration on *An. funestus* underscores the potential of this low-cost, reagent-free technique for widespread use on all the major Afro-tropical malaria vectors. Future research should demonstrate how such machine-derived age classifications in field-collected mosquitoes correlate with malaria in human populations.

**Graphical Abstract:**

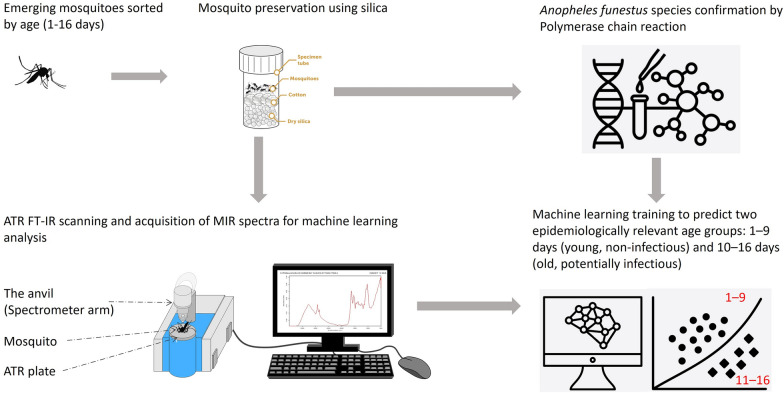

## Background

Despite significant investments in malaria control and research, there were still an estimated 249 million malaria cases and 619,000 deaths in 2021 globally, a significant majority of which occurred in sub-Saharan Africa [1]. Other than the poor economic conditions and weak health systems, the continued high burden of malaria in Africa is attributable to key biological threats, notably malaria parasite resistance to drugs [2–4], vector resistance to insecticides [5, 6], increasing occurrence of malaria parasites evading detection by rapid diagnostic tests [7–11], and disruptions from major disease outbreaks such as Ebola and COVID-19 [12–14]. Effective vector control, primarily with insecticide-treated nets (ITNs) and indoor residual spraying (IRS), has been the most important component of malaria control in Africa [15]. However, its continued effectiveness requires active innovation to address the current threats, and improved understanding of the major vector species in different settings.

*Anopheles funestus* is one of the four main malaria vector species in sub-Saharan Africa, the others being *An. gambiae*, *An. arabiensis* and *An. coluzzii*, and it is also one of the most widespread [16–19]. *Anopheles funestus* is particularly important in East and Southern Africa, where it is becoming the dominant malaria vector. For example, in parts of Tanzania, *An. funestus* is reported to be responsible for 86–97% of all new malaria infections [17, 20–22]. Its dominance is due to multiple factors, including (i) being highly anthropophilic, and thus preferring to bite humans over other vertebrates [17, 23], (ii) being highly endophilic, i.e. preferring to bite inside human dwellings rather than outside [24], (iii) having significantly higher survival rates than other species [25], (iv) being resistant to commonly used insecticides [17, 18, 26] and (v) preferentially breeding in perennial habitats with year-round productivity [27]. Given its importance and dominance in malaria transmission systems, vector surveillance programs in the respective countries should be designed with special attention to this vector species.

Besides evaluating biting densities and *Plasmodium* infection rates, accurately determining the age and survival of *An. funestus* is crucial for monitoring transmission dynamics and assessing the effectiveness of vector control interventions such as ITNs and IRS. Dissection of mosquito ovaries is still the main entomological technique for estimating the age of vector populations [28]. The dissections are usually performed under light microscopes to assess the reproductive history, specifically the parity status, of the mosquitoes. This involves observing whether the ovaries contain coiled tracheolar skeins (indicating non-parous mosquitoes) or stretched-out tracheoles (indicating parous mosquitoes). Non-parous mosquitoes are considered young in this case, whereas parous mosquitoes are considered old and may carry the malaria parasites, having had multiple blood-feedings [28]. Unfortunately, these dissections tend to be laborious and time-consuming, especially when dissecting large numbers of mosquitoes, and are impractical on a large scale.

Furthermore, the reliability of mosquito dissections is limited by their reproductive history. For instance, a female mosquito can have more than one blood meal but still not oviposit, a scenario known as gonotrophic discordance or pre-gravid blood meal [29]. Moreover, since the gonotrophic cycles of *Anopheles* mosquitoes can be as short as 2–3 days under optimal climatic conditions [30, 31], it is possible for parous mosquitoes to be relatively young, and in rare cases for nulliparous mosquitoes to be several days old due to the scarcity of blood meals (e.g. when ITN coverage and usage is high). Therefore, using parity alone to distinguish between epidemiologically distinct age categories of adult mosquitoes, especially in the context of malaria transmission, which requires 10–14 days of incubation [32], is not always realistic.

All these concerns suggest the need for alternative age-grading techniques that are easy to perform cheaply at scale and can provide accurate representations of epidemiologically important mosquito age categories and populations. The alternative mosquito age-grading methods currently include the analysis of cuticular hydrocarbon patterns using gas chromatography [33] and gene transcription [34–36]. Near-infrared spectroscopy (NIRS) (12,500 cm^−1^ to 4000 cm^−1^ frequencies) [37], which involves passing infrared light through a mosquito sample to measure the absorbance or reflectance of the organic compound functional groups, has also been used to estimate ages for various mosquito species of both laboratory-reared and wild-collected mosquitoes [38–44].

More recently, mid-infrared spectroscopy (MIRS) has been used to predict and estimate mosquito age, recording the biochemical composition of mosquito samples at longer wavelength frequencies [45–47]. In addition, machine learning (ML) techniques, including convolutional neural networks, have been utilized to differentiate MIRS spectra associated with distinct mosquito ages and species in both laboratory and wild mosquitoes [46, 47]. The infrared-based systems have so far been successful for various applications on three of the four main African malaria vectors (i.e. *An. gambiae* sensu stricto, *An. arabiensis* and *An. coluzzii* [46]), but have yet to be demonstrated for *An. funestus*. The goal of this study was therefore to test whether a similar ML-MIRS approach could classify adult female *An. funestus* mosquitoes derived from wild-caught larvae into two epidemiologically relevant age categories: young (0–9 days old, too young to have mature *Plasmodium* sporozoites in their salivary glands) and old (10 days or older, potentially carrying mature *Plasmodium* sporozoites given the right climatic conditions), factoring in a parasite incubation period of 10–14 days.

## Methods

### Mosquito collection

Third and fourth instar mosquito larvae were collected from known aquatic habitats of *An. funestus* in five different villages in south-eastern Tanzania, namely Tulizamoyo (8.3669°S, 36.7336°E), Kilisa (8.3721°S, 36.5584°E), Lupiro (8.38333°S, 36.66667°E), Ikwambi (7.9833°S, 36.8184°E) and Ruaha (8.9068°S, 36.7185°E). The larvae were transported to the vector biology laboratory (VectorSphere) at the Ifakara Health Institute for further rearing. The larvae were kept in water from their natural breeding habitats and were fed TetraMin^®^ fish food.

Once they pupated, the pupae were separated from the larvae and placed in emergence cages. The emergent adult mosquitoes were maintained at 26–28 °C, 70–85% relative humidity and a 12:12 h light/dark photoperiod, on a 10% sugar solution diet.

### Mosquito preservation and scanning

Female adults were collected and individually preserved according to their age, from 1 to 16 days old. A total of 2084 mosquitoes were collected. The female mosquitoes were killed using chloroform and subsequently stored in separate 1.5-ml microcentrifuge tubes containing silica gel for desiccation. The heads and thoraces of the individual female mosquitoes were scanned using an attenuated total reflection–Fourier-transform infrared spectrometer (ATR FT-IR) to obtain mid-infrared spectra with a resolution of 2 cm^−1^ at 4000–400 cm^−1^ frequencies as described previously, complete with background spectral calibration [45, 48, 49]. For each sample, 16 sample scans were averaged to obtain the primary output spectrum [46].

### Mosquito identification

Although the field collections had been performed in known *An. funestus* habitats, it was necessary to confirm the identity of the mosquitoes and eliminate any unwanted species. This was accomplished primarily by morphology-based taxonomy using keys of Afro-tropical *Anopheles* [50] but was complemented by polymerase chain reaction (PCR) identification to sort between sibling species in the *An. funestus* group. Wild *An. funestus* complex DNA was extracted from the two legs of adult female mosquitoes. The two legs of an individual *An. funestus* mosquito were placed separately in 1.5-ml microcentrifuge tubes, followed by 20 µl of TE (Tris-EDTA) buffer, and incubated at 95 °C for 15 min. PCR was then used to differentiate *An. funestus* from other sibling species, using species-specific primers targeting the non-coding internal transcribed spacer (ITS2) region using the protocol described by Koekmoer et al. [51]. The PCR reaction was performed in a 25 µl volume, consisting of a PCR mixture of 2.5 µl 10× reaction buffer, 25 mM MgCl_2_, 10 pmol/µl of each primer, 8 mM of each dNTP, 5 units of thermostable Taq DNA polymerase and 3 µl of DNA template. The PCR products were analysed by electrophoresis in 2.5% agarose gel stained with classic view DNA dye for visualization of DNA bands. Only *An. funestus* mosquitoes were considered for further analysis, and any other species were discarded.

### Machine learning

Mosquito spectra with low intensity, abnormal background or atmospheric interferences (with water vapor and carbon dioxide) were discarded [45]. The data from the remaining spectra (*N* = 2084) were processed and analysed in Python 3.9 using scikit-learn [52] and TensorFlow 2.0 [53, 54]. The data were rescaled using the StandardScaler algorithm, with a mean of 0 and a standard deviation of 1.

Using the StratifiedShuffleSplit algorithm, the dataset was split into training (*n* = 1875) and test/unseen (*n* = 209) sets. To train the supervised ML models, *An. funestus* ages were used as training labels. *Anopheles funestus*, ranging from 1 to 16 days old, were divided into two epidemiologically relevant age categories, taking into consideration the incubation period of malaria parasites of 10–14 days [32]. The first group included *An. funestus* that were between 1 and 9 days old and were considered young and incapable of transmitting malaria (i.e. non-infectious age group). The second group included *An. funestus* that were between 10 and 16 days old and were considered old enough to be capable of transmitting malaria given the right environmental conditions (i.e. potentially infectious).

Multiple standard ML classifiers, including *k*-nearest neighbours (KNN), logistic regression (LR), support vector machine (SVM), random forest (RF) and extreme gradient boosting (XGBoost), were compared to determine which model predicted the data with the highest classification accuracy. The best-performing model was further optimized by fine-tuning its hyperparameters. The top 100 spectral features (wavenumbers) with the most influence on the model predictions were identified and utilized to reduce the dimensionality of the spectra data, followed by retraining of the best ML classifier.

Moreover, two multi-layer perceptron (MLP) models were trained by reducing the dimensionality of the spectra data using different inputs: (1) the top 100 features extracted from the best-performing ML classifier, and (2) principal components using the scikit-learn library. Both MLP models had six fully connected layers, each containing 500 neurons, to enable the model to learn from the network's weights, as demonstrated previously [47]. To prevent overfitting, a dropout layer with a rate of 0.5 was used, and early stopping was implemented when the validation loss could no longer improve after 400 iterations [55, 56]. The model performance was evaluated using *k*-fold cross-validation (*k* = 5) to ensure an unbiased assessment of the standard ML and MLP models, as described previously [37].

To assess the ability of the optimized models to identify all positive instances and avoid false negatives, the recall score (i.e. sensitivity or true positive rate) was estimated as the ratio of correctly age-classified *An. funestus* to the total number of *An. funestus* in the respective age category in the dataset. Moreover, to measure the ability of the models to avoid false positives, the precision score (i.e. the positive predictive value) was estimated as the ratio of correctly age-classified *An. funestus* to the total number of predicted positive instances of the respective age categories. Lastly, we calculated the *F1* score, which balances both precision and recall scores by giving equal weight to both measures. This score provides a single value that represents the overall performance of the model in terms of its ability to correctly classify positive and negative cases. A higher *F1* score signifies better model performance, where a maximum value of 1 represents flawless precision and recall.

## Results

### Predicting *An. funestus* age classes using standard ML models

**Fig. 1:**
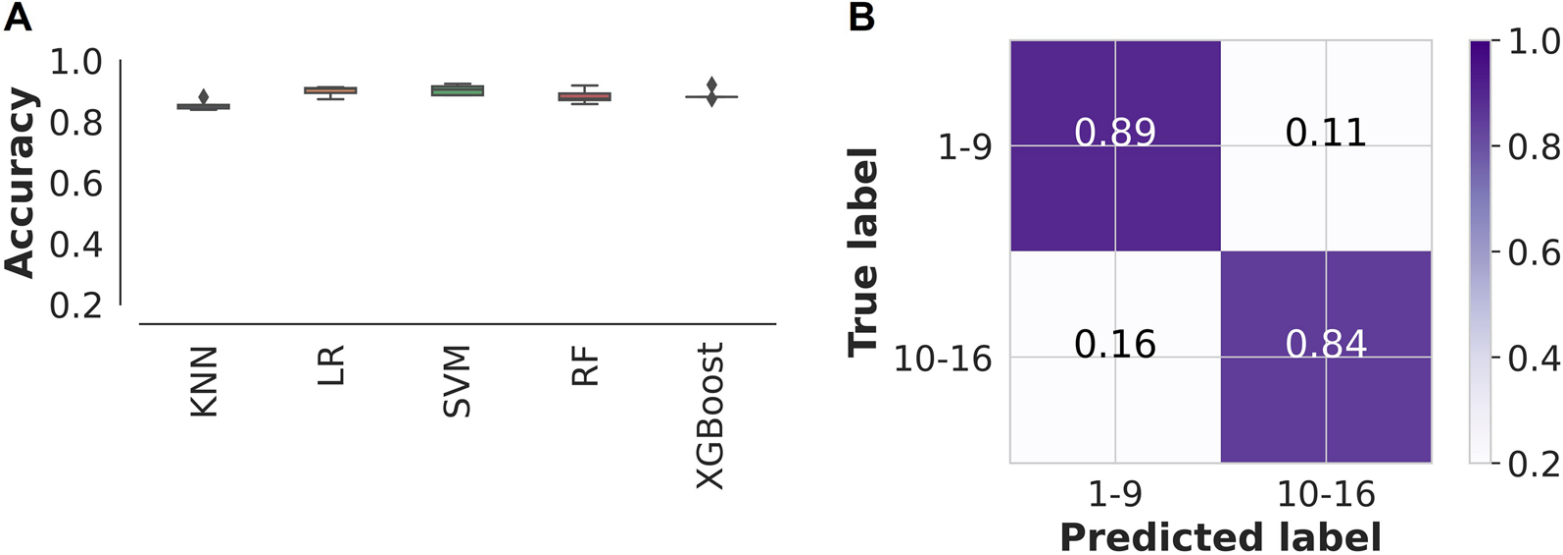
Machine learning prediction of An. funestus age classes. **A** Comparison of standard ML classifiers in predicting *An. funestus* age classes; *KNN* *k*-nearest neighbours, *LR* logistic regression, *SVM* support vector machine, *RF* random forest, *XGBoost* gradient boosting, *MLP* multilayer perceptron. **B** Confusion matrix for predicting the age class of *An. funestus* using XGBoost on an unseen dataset, results for the ML trained with all spectral features

In the initial comparison of standard ML models, XGBoost emerged as the best classifier with the highest prediction accuracy and lowest standard deviation, achieving 84% accuracy (Fig. 1A). After optimizing the parameters, the XGBoost model was able to classify spectra that were previously unseen with an overall accuracy of 87%. It achieved accuracy of 89% and 84% for young (1–9 days old) and old (10–16 days old) *An. funestus* females, respectively (Fig. 1B). The recall scores (i.e. sensitivity or true positive rates) of this model were 0.89 and 0.84 for the young and old mosquitoes, respectively, while its precision scores (i.e. the positive predictive value) were 0.87 for both age categories (Table 1).

**Table 1:** Precision, recall and *F1* score of XGBoost and multi-layer perceptron (MLP) models for predicting age categories of *An. funestus*

Model	Age classes (days)	Precision	Recall	*F1*-score	No. of test samples
XGBoost 1	1–9	0.87	0.89	0.88	113
	10–16	0.87	0.84	0.86	96
XGBoost 2	1–9	0.88	0.92	0.90	113
	10–16	0.90	0.85	0.88	96
MLP 1	1–9	0.95	0.95	0.95	113
	10–16	0.94	0.94	0.94	96
MLP 2	1–9	0.94	0.93	0.93	113
	10–16	0.92	0.93	0.92	96

XGBoost 1: Trained with all MIRS wavenumbers (*n* = 1665), XGBoost 2: Trained with spectral features extracted based on feature importance summaries (*n* = 100), MLP 1: Trained with spectral features extracted based on feature importance summaries (*n* = 100), MLP 2: Trained with principal component analysis (PCA) as a dimensionality reduction technique

**Fig. 2:**
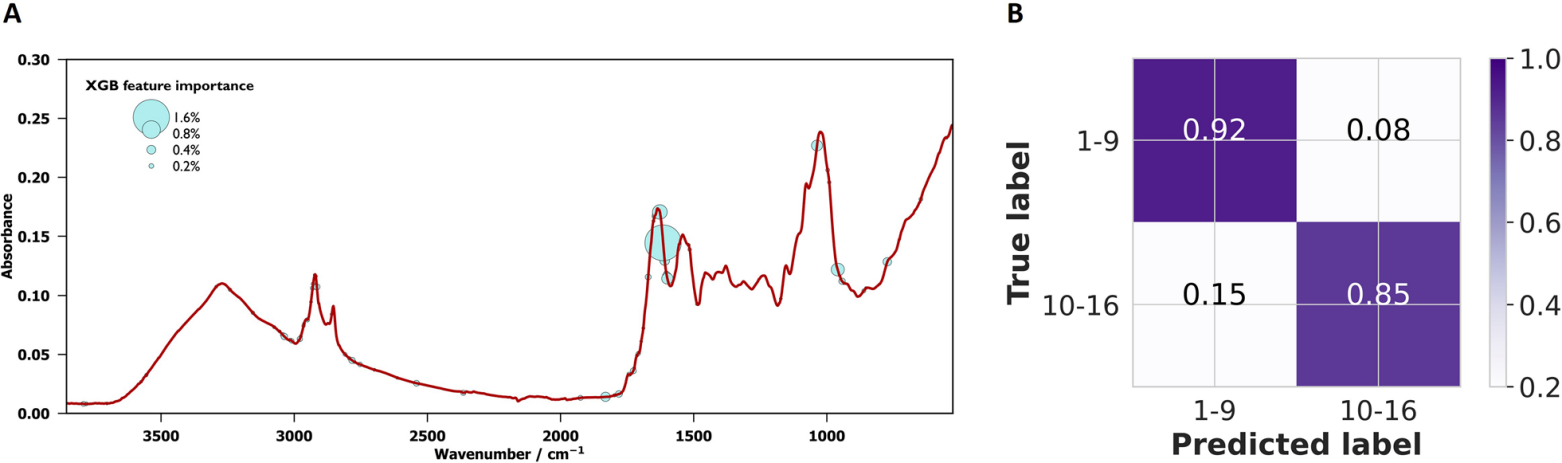
**A** Relative importance of XGBoost features that have the most influence in predicting the age classes of *An. funestus*. **B** Confusion matrix for predicting the age class of *An. funestus* using XGBoost on an unseen dataset; the results for the ML retrained with important features/wavenumbers (*n* = 100) identified by the initial XGBoost model

From the initial XGBoost model, we identified the spectral features that were most important for the prediction. This analysis aimed to reduce the number of training features and enhance the accuracy of the model during retraining (Fig. 2A). When the XGBoost classifier was retrained with the top 100 features, the classification accuracy increased to 89%, correctly predicting young and old *An. funestus* females with 92% and 85% accuracy, respectively (Fig. 2B).

### Prediction of *An. funestus* age classes using MLP models

**Fig. 3:**
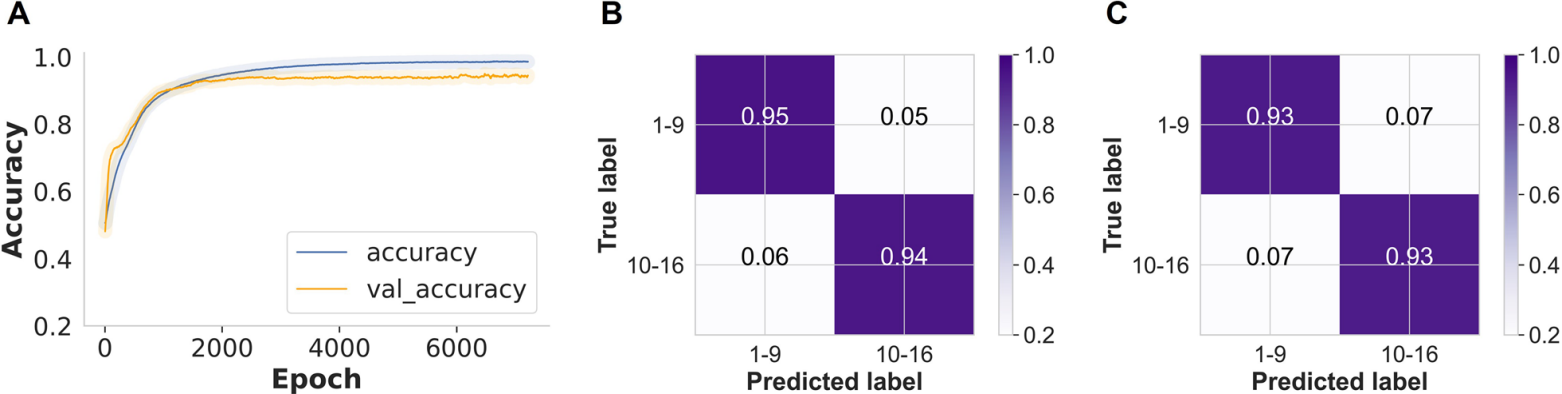
**A** MLP training and validation accuracy for *An. funestus* age classes as training time increases (epoch; number of iterations over the entire dataset during the training process, i.e. seconds/iterations). Confusion matrix for predicting the age class of *An. funestus*. **B** The results for the MLP trained with important features/wavenumbers (*n* = 100) identified by XGBoost. **C** The results for the MLP method trained with eight principal components

We explored the possibility of improving the accuracy by training the MLP classifier using the important wavenumbers (*n* = 100) identified in the XGBoost predictions. As a result, the MLP achieved an improved accuracy of 94.5% in the unseen test data (Fig. 3A), correctly distinguishing between young and old *An. funestus* females with accuracy of 95% and 94%, respectively (Fig. 3B).

Lastly, in a previous study, we presented evidence that employing principal component analysis (PCA) with eight components effectively reduces the dimensionality of the spectral data [47]. This reduction in dimensionality not only preserved a substantial portion of the data variability but also mitigated overfitting while enhancing the signal-to-noise ratio. By utilizing a reduced set of features, we trained the MLP model to improve its predictive performance [47]. In the present study, when PCA was utilized to reduce the dimensionality of the spectra data, the MLP classifier achieved an overall accuracy of 93% for both young and old *An. funestus* mosquitoes (Fig. 3C).

## Discussion

*Anopheles funestus* mosquitoes are currently the major vector of malaria transmission in Tanzania, accounting for over 80% of malaria transmission [17, 20–22]. *Anopheles funestus* tends to have better survival rates [25], and is generally a slow-growing mosquito, which adds to the challenge of studying its demographic characteristics and how these might influence pathogen transmission. Here, we present a rapid age-grading technique that has the potential to replace traditional methods such as ovarian dissections, which are time-consuming and challenging to apply on a large scale. Using 2084 spectral data points, we trained ML models that classify the epidemiologically relevant age groups of *An. funestus* mosquitoes reared from wild larvae using water from the same habitats, but under laboratory conditions. The models correctly distinguished between the young *An. funestus* females (1–9 days old) and the older ones (10–16 days old) based on the MIR spectra indicative of the varying biochemical composition of the mosquito cuticles [57]. While this was the first demonstration of the effectiveness of this technique for predicting the age of *An. funestus* mosquitoes, the approach of combining infrared spectra and ML models has been widely demonstrated for predicting different indicators, including age, blood meals, infection status and insecticide resistance profiles of other *Anopheles* species [46–48]. If validated on field-collected adults, these findings could be a step towards wider applications of this approach for malaria vector surveillance in settings with different vector species.

In settings such as rural south-eastern Tanzania where *An. funestus* is the dominant malaria vector [17, 20], it is particularly important that vector surveillance programs are expanded to include this vector species. Indeed, the successful demonstration of this technique on *An. funestus*, which is one of the most efficient and also most widespread malaria vectors in Africa [58], expands the utility range of this technique for a much broader application for malaria vector surveys in different parts of Africa.

One of the key concerns regarding previous applications of MIRS-ML-based approaches for entomological assessments is that, with the exception of some cases [46], these methods have been rarely validated for wild-caught malaria vectors in field settings. Here, *An. funestus* mosquitoes were collected as larvae from various villages and breeding habitats, to account for genetic variation, variation in larval food sources and microbiome, and to maintain some characteristics of the natural ecosystems. The success of this analysis and the high accuracy obtained may therefore be indicative of the potential of the approach for predicting key mosquito attributes in field settings. However, it is unknown whether specific climatic factors could influence the prediction and generalizability of the MIRS-ML approach. Future studies should therefore test the generalizability of this approach across different populations of wild mosquitoes.

This study classified mosquitoes only as young (1–9 days old) or old (10–16 days old) and did not attempt to classify them at specific chronological ages because the sample size was not large enough to test it. However, the chosen age classes represent the typical epidemiological distinction relevant to the transmission of malaria parasites, which, under standard climatic conditions, requires a vector to be at least 10 days old [32]. However, it may fail to capture variations in MIR spectra or the small biochemical changes that occur within a mosquito cuticle after each ageing day (such as chronological age from 1 up to 16) [45]. Moreover, it has been demonstrated that calibrating ML models based on physiological age (which considers key life cycle processes such as blood-feeding and oviposition) may be more useful than simply relying on chronological age classifications [38, 59]. In our study, mosquitoes were all sugar-fed, and therefore physiological age was not assessed. Future efforts should assess key differences in these approaches and evaluate models trained on biological age and chronological age to determine which ones are most practical and most generalizable. An obvious next step is therefore to investigate any correlations that might exist between the machine-classified age categories and the epidemiology of malaria in human populations.

To improve the classification accuracy of our model, the XGBoost feature importance was relied upon to reduce the number of spectral features from 1665 to 100. This dimensionality reduction significantly lowered the noise and redundant features in the MIR spectra data. The important features were mostly associated with proteins, with the most influential peak (1700 cm^−1^) being the band associated with the amide bond from proteins. The region around 3000 cm^−1^, which is also related to proteins, was also found to be important in the model prediction. This implies that the model is learning from protein-based biological traits that vary depending on the age of the mosquito [46]. Moreover, when PCA was used to reduce the dimensionality of the spectra from 1665 features to eight principal components [47], the prediction accuracy matched that of the MLP model trained with the top 100 biological features as identified from the XGBoost model. This suggests that ML models may perform better when trained with fewer features that explain more variation in the data, rather than many redundant features that introduce noise into the model. Moreover, as observed previously, reducing the dimensionality of the spectra data reduces the computational resources needed to train ML models [47].

Future research should investigate the effects of rearing wild *An. funestus* larvae in the insectary on the predictive accuracy of the MIRS-ML approach for mosquito age-classification, as this could impact the generalizability of the findings.

## Conclusions

This study demonstrates the classification of adult female *An. funestus* into distinct and epidemiologically relevant age categories using a MIRS-ML approach. In conjunction with prior research conducted on other *Anopheles* mosquitoes, this study suggests that the applicability of this approach can be extended to evaluate various entomological attributes in *An. funestus*. The MIRS-ML approach proves to be quick and cost-effective, and has the potential to significantly enhance *An. funestus* surveillance efforts, thereby contributing valuable insights to national malaria control programs, particularly in resource-constrained settings where this vector is highly prevalent. Nonetheless, further research is needed to validate the MIRS-ML approach in field conditions, using adult *An. funestus* populations and other vector species within malaria-endemic communities, and to examine how the machine-classified age categories correlate with the epidemiological strata of malaria in human populations.

## Data Availability

The mid-infrared spectral datasets generated and analysed during the current study, as well as code for the analyses, is available at [GitHub].

## Abbreviations

MIRS: Mid-infrared spectroscopy
NIRS: Near-infrared spectroscopy
PCR: Polymerase chain reaction
ITNs: Insecticide-treated nets
MIRS-ML: Mid-infrared spectroscopy and machine learning
